# Anti-Claw Procedure with Modified Dynamic Flexor Digitorum Superficialis Transfer for Ulnar Nerve Palsy: Our Experience

**DOI:** 10.5704/MOJ.2211.012

**Published:** 2022-11

**Authors:** MA Iqbal, I Khan, SSU Rehman, MSA Beg

**Affiliations:** 1 Department of Plastic Surgery, Liaquat National Hospital and Medical College, Karachi, Pakistan; 2 Department of General Surgery, Liaquat National Hospital and Medical College, Karachi, Pakistan

**Keywords:** anti-claw procedure, ulnar nerve palsy, flexor digitorum superficialis transfer, ulnar claw hand

## Abstract

**Introduction:**

The ulnar nerve palsy is a distressing injury, resulting in clawing of hand. The cause of clawing is due to paralysis of the interosseous muscles in the presence of functioning long extensor and long flexors of fingers. Various methods have been proposed to correct this deformity which include both static and dynamic procedures. In this study, we share our experience with flexor digitorum superficialis tendon transfer using Zancolli’s modification for anti-claw correction.

**Materials and methods:**

It was a retrospective case series study. A record of 53 patients was included in the study, during a period between June 2013 to July 2017 with ulnar nerve palsy. The procedure done was flexor digitorum superficialis tendon transfer as dynamic anti-claw procedure. The follow-up period was three months. The outcomes assessed were grip strength by using sphygmomanometer and active range of motion of fingers assessed by fingers tips touching the palm.

**Result:**

Fifty-three patients were included out of them, there were fifty males and three females. The mean age was 28±10 years. All patients underwent flexor digitorum superficialis transfer for ulnar claw hand. A total of 84.9% patients have good grip strength and 83% showed good active range of motion.

**Conclusion:**

Flexor digitorum superficialis tendon transfer is found to be effective, reliable and reproducible technique in ulnar nerve palsy where patient need grip strength, good range of motion and acceptable hand function for daily routine work.

## Introduction

Ulnar nerve palsy is a devastating injury resulting in clawing caused by paralysis of the intrinsic muscles in the presence of functioning long extensor and flexor of fingers. Claw hand deformity due to loss of active IPJ extension and MCPJ flexion, which prevents the patient from cupping the hand around an object. Different modifications of FDS transfer have been described^1,2^.

Stiles and Forrester-Brown first describe FDS transfer; they transfer one slip of FDS to extensors digitorum of each proximal phalanx. Bunnel modified by rerouting both FDS sling through lumbrical canal and suture them to radial and ulnar band of extensors. Littler modified this using only one FDS sling and divide it into two. Zancolli lasso altered it by passing FDS slip to A1 pulley. Omer modified it by passing FDS slip to A2 pulley.

In this study we shared our experience of treating ulnar claw hand by choosing a dynamic anti-claw procedure. We have used the flexor digitorum superficialis tendons of middle and ring fingers dividing into two slips. This modification was briefed by Pillukat^3^.

## Materials and Methods

It was a case series study. This study had conducted in Liaquat National Hospital and Medical College after taking approval from Ethical Review Committee. It had shown a record of 53 patients which were assessed between June 2013 to July 2017 with ulnar nerve palsy in Department of Plastic and Reconstructive surgery.

All patients with flexor digitorum superficialis transfer were included in the study via simple consecutive sampling technique. All patients had been assessed by the senior author in OPD. The consent had been taken from patients before inclusion in study. The procedure had been performed by the senior author only. The minimum follow-up period was 3 months and maximum was 12 months.

**Fig. 1.**
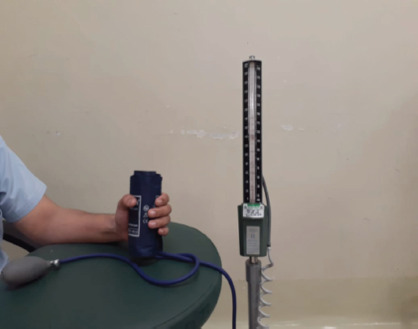
Demonstrating hand grip strength using sphygmomanometer.

We standardised the follow-up at three months as further follow-up did not show any change in outcome of study. The outcomes assessed were grip strength of hand and active range of motion of fingers. The grip strength was assessed by using the sphygmomanometer as it is easily available, and results are reproducible and reliable (Fig. 1). (Mercury base apparatus, Certeza ®, size of blood pressure cuff; 25.4/40.6cm) with rolled blood pressure cuff set at 20mm of Hg of pressure; considered as base line^4^. Based on a pilot study, we identified the following measurement by using sphygmomanometer and categorised accordingly.

Patient asked to squeeze rolled pressure cuff and deviation of reading noted. Good grip strength of hand was considered if >200mmHg of pressure was achieved on sphygmomanometer; Fair grip strength was considered if the reading was between 100 to 199mmHg of pressure and <100mmHg of pressure was considered as poor grip strength.

The active range of motion of fingers touching to palm was categorised as good range of motion, if fingertips touched mid palm. If fingertips touched proximal palmar crease, then considered as fair range of motion. If finger tips not touching the palm then it was considered as a poor range of motion^5^.

Statistical Package for Social Science (SPSS) version 21.0 used for descriptive analysis and correlations, with confidence interval of 95%. Level of significance (p-value) was taken as less than or equal to 5%.

**Fig. 2.**
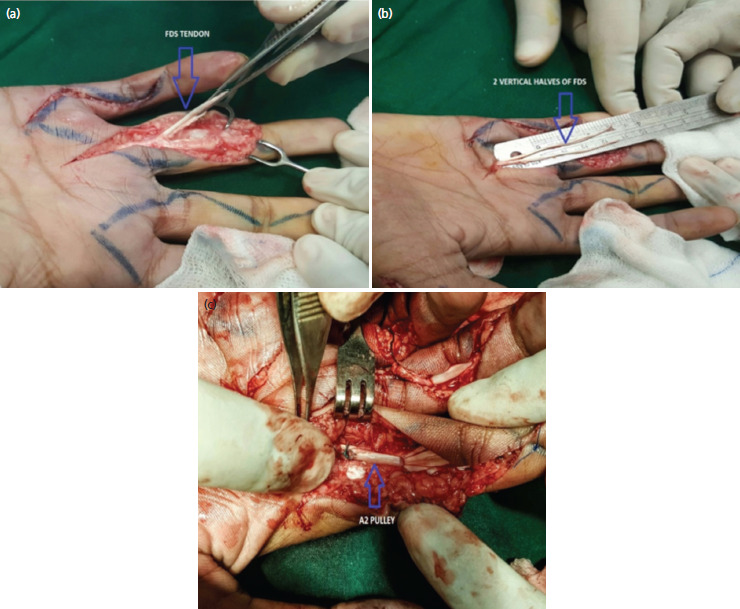
(a) Exploration of FDS tendon, (b) splitting of each slip of FDS tendon into two vertical halves, (c) tendon looped to A2 pulley.

Chi square and Fisher exact test were used to find the association between the categorical variables. The operative technique involved the detachment of one slip of flexor digitorum superficialis from each middle and ring finger and the other slip is preserved resulting in the preservation of middle and ring finger, respectively^3,4^ (Fig. 2a).

Each harvested slip split into two vertical halves (Fig. 2b). Each divided slip passed through A2 pulley of index, middle, ring and little finger. The slip sutured around A2 pulley in looped manner with non-absorbable suture (Fig. 2c). The hand was kept in cast for four weeks and physiotherapy within cast started from 2nd post-operative day to prevent adhesions.

## Results

**Fig. 3.**
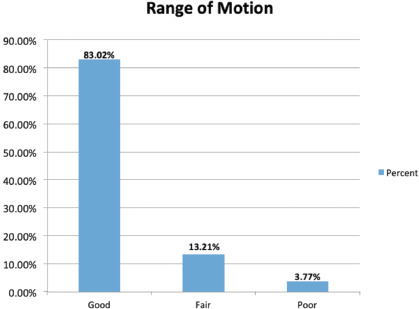
Demonstrates range of motion after tendon transfer.

**Fig. 4.**
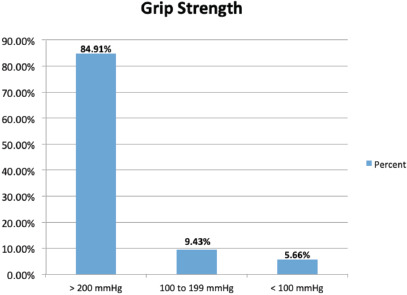
Demonstrates grip strength after tendon transfer.

Total of 53 patients included in our study, 3 females and 50 males. They were surgically treated for ulnar claw hand deformity. The average age of patient was 28±10 (21 to 39). Final follow-up performed after an average of three months. Three grades had been used to record the result of active range of motion, which showed in (Table I) (Fig. 3). Good active range of motion were found in 83.02%, fair active range of motion were found in 13.21% and poor active range of motion in 3.77% patient. Grip strength was assessed by using sphygmomanometer. A total of 84.91% patient able to squeeze above 200mmHg of pressure, 9.43% able to squeeze in range of 100 to 199mmHg of pressure, only 5.66% show poor result with grip strength in less than 100mmHg of pressure (Table II) (Fig. 4).

**Table I:** Demonstrating frequency of range of motion

Range of motion	Frequency	Percent (%)
good- finger tip touches mid palm	44	83.02
fair- finger tip touches proximal palmer crease	7	13.21
poor- finger tip not touching palm	2	3.77
Total	53	100.0

**Table II:** Demonstrating frequency of grip strength

Grip strength	Frequency	Percent (%)
> 200 mmHg	45	84.91
100 to 199 mmHg	5	9.43
< 100 mmHg	3	5.66
Total	53	100

There was a highly significant association of grip strength (p<0.001) with active range of motion. There was statistically insignificant association of the mechanism of injury (p=0.168) with the grip strength and the active range of motion (p=0.525).

There was statistically insignificant association of gender (p=0.272), mechanism of injury (p=0.067), range of motion (p=0.188) and grip strength (p=0.118) with the age group. There was statistically significant association of the mechanism of injury (p=0.038) with the gender while there was statistically insignificant association of the range of motion (p=0.435) and the grip strength (p=0.394) with the gender.

## Discussion

Ulnar nerve palsy resulted in claw hand deformity and failure of synchronous finger motion which is referred to as claw hand disability. It is due to loss of function of all the interossei, and the ulnar two lumbricals creates loss of metacarpophalangeal flexion and weakness of the extensor mechanism due to loss of lumbrical pull on the lateral bands. The action of fourth and fifth long finger flexor digitorum profundus and flexor digitorum superficialis moves the distal interphalangeal joints and the proximal interphalangeal joints into flexion posture, whereas the unopposed pull of the extrinsic extensors creates an extension movement at the metacarpophalangeal joints, thus creating the clawed appearance.

If the ulnar claw hand is not addressed, then the third and second digits may claw over period of time as their lumbricals alone may be unable to match the power of the extrinsic extensors^6^. A number of dynamic procedures have been explained to improve claw hand deformity and to prevent hyperextension of metacarpophalangeal joint.

The literature has explained techniques including Zancolli lasso, Modified Stiles-Bunnell and Brand procedures^7-9^. Most commonly used procedures are Zancolli lasso and modified Stiles-Bunnell tendon transfers which involves volar flexor digitorum superficialis transfers and do not need tendon grafting^10-11^. Clawing of the ring and little fingers showed good results after tendon transfer surgeries, improvement in hand functions including grip strength, pinch and even flat hand function^11,12^.

In our study, we used the technique originally described by Pillukat *et al*^3^, as using flexor digitorum superficialis tendons of middle and ring finger. We had found good results not only for grip strength but also for range of motion. We had found that 45 patients (84.9%) have showed good grip strength of >200 mmHg measured via sphygmomanometer.

However, the mechanism of injury had not been compared before with grip strength and range of motion in other previous studies^1,6^ as we had compared in our study and showed the association. It can be stated that there were no equal number of distributions in males and females so significant association with gender could not be presented.

The other variable we had assessed was active range of motion in hand by evaluating flexion at the level of wrist. Of the 52, 44 (83%) patients had showed flexion of fingers to distal palmar crease considering it to be good range of motion. Seven individuals (13.2%) showed fair range of motion till mid palmar crease and only two (3.8%) had poor results with palmar flexion to proximal palmar crease.

We had found comparable results to the original technique described by Pillukat as grip strength was on average 58±28% in comparison to our study as 84.9±0.532%. Alkhooly *et al*^13^ in their study had excellent to good results in all patients (100%) showing range of motion using modified Bunnell’s technique^13^.

There were various advantages of this procedure including easy to perform, reproducible, short learning curve, better cosmetic outcomes and easier to retrain the muscles. The only prerequisites for procedure include availability of flexor digitorum superficialis along with working flexor digitorum profundus.

The limitation of study included were retrospective study with limited number of patients, single centre study and have no equal number of gender distribution. There was a need to include an objective scale (e.g., DASH Score).

## Conclusion

In ulnar nerve palsy where the patient needs grip strength and acceptable hand function for daily routine work, modified flexor digitorum superficialis tendon transfer is found to be effective, reliable and a reproducible option.
